# *MiR-663*, a MicroRNA Linked with Inflammation and Cancer That Is under the Influence of Resveratrol

**DOI:** 10.3390/medicines5030074

**Published:** 2018-07-09

**Authors:** Jean-Jacques Michaille, Victoria Piurowski, Brooke Rigot, Hesham Kelani, Emily C. Fortman, Esmerina Tili

**Affiliations:** 1 BioPerox-IL, UB-INSERM IFR #100, Faculté Gabriel, Université de Bourgogne-Franche Comté, 21000 Dijon, France; Jean-Jacques.Michaille@u-bourgogne.fr; 2 Department of Biology, Franklin College of Arts and Sciences, University of Georgia, Athes, GA 30602, USA; victoria.piurowski@uga.edu; 3 Department of Cancer Biology and Genetics, Wexner Medical Center, The Ohio State University, Columbus, OH 43210, USA; rigot.10@buckeyemail.osu.edu; 4 Department of Anesthesiology, Wexner Medical Center, The Ohio State University, Columbus, OH 43210, USA; Hesham.Kelani@osumc.edu (H.K.); fortman.64@buckeyemail.osu.edu (E.C.F.)

**Keywords:** resveratrol, *miR-663*, inflammation, cancer, cardiovascular disease

## Abstract

Resveratrol (trans-3,5,4′-trihydroxystilbene, RSV) is a non-flavonoid dietary polyphenol with antioxidant, anti-inflammatory and anti-cancer properties that is primarily found in red berries. While RSV displays many beneficial effects in vitro, its actual effects in vivo or in animal models remain passionately debated. Recent publications suggest that RSV pleiotropic effects could arise from its capability to regulate the expression and activity of microRNAs, short regulators themselves capable of regulating up to several hundreds of target genes. In particular, RSV increases microRNA *miR-663* expression in different human cell lines, suggesting that at least some of its multiple beneficial properties are through the modulation of expression of this microRNA. Indeed, the expression of microRNA *miR-663* is reduced in certain cancers where *miR-663* is considered to act as a tumor suppressor gene, as well as in other pathologies such as cardiovascular disorders. Target of *miR-663* include genes involved in tumor initiation and/or progression as well as genes involved in pathologies associated with chronic inflammation. Here, we review the direct and indirect effects of RSV on the expression of *miR-663* and its target transcripts, with emphasise on *TGFβ1*, and their expected health benefits, and argue that elucidating the molecular effects of different classes of natural compounds on the expression of microRNAs should help to identify new therapeutic targets and design new treatments.

## 1. Introduction

Recent years have brought an increasing number of publications describing the potentials of plant natural products to be used for the treatment of human pathologies. Among those molecules, Resveratrol (trans-3,5,4′-trihydroxystilbene, RSV), which is produced by plants in defense against the pathogen *Botrytis cinerea* [1], is in particular found in the skins of black and red berries. RSV presents strong antioxidant, anti-inflammatory and anti-cancer properties [2,3]. While most of these properties have been demonstrated using cell cultures, RSV precise molecular effects in vivo have remained controversial, in particular because the bioactivity of this compound is limited by the fact that it is rapidly metabolized [4]. Finally, whether resveratrol alone is beneficial to the health, or whether resveratrol metabolites actually participate in delivering beneficial effects, also remains a matter of debate (discussed in [5]).

Pre-clinical studies, however, are presently being conducted to determine the true therapeutic potentials of RSV in the treatment of cancer and cardiovascular diseases [3,4,5,6,7,8]. On the one hand, several promising results have been reported. For example, the potentials of RSV as a phytoestrogen, an inhibitor of aromatase activity and an adjuvant has been explored in the context of breast cancer [3]. Other studies have been conducted in patients with colorectal, multiple myeloma neuroendocrine tumors, with variable results [7,8]. Beneficial effects of RSV on patients with neurologic, metabolic, and cardiovascular pathologies have also been reported [6,9,10,11,12]. Thus, in animal models, resveratrol has been shown to be anti-hypertensive, to modulate the levels of HDLs and LDLs in rats subjected to high fat diet, to reduce myocardial ischemia and ischemia-reperfusion injury, and to reduce cardiac hypertrophy; however, several studies have reported no RSV effect in each type of pathologies (reviewed by [5]).

The biggest challenge finally rests on determining the molecular pathways through which RSV acts in different pathological contexts, determining the optimal RSV dose, the best mode of RSV delivery, and how to increase the bioavailability of RSV in different tissues (discussed in [6]). Based on studies conducted in animal models such as swine, mouse or monkeys, the absorption of regular, low doses of RSV seems to bear promising potentials for preventive therapeutics [6]. Transferring these results in human, however, remains particularly challenging, in particular when it comes to measuring RSV protecting effects in healthy subjects, given in particular the impossibility of conducting long or very long-term studies, that, beyond considerable cost, carry high risks of bias [6]. Thus, while beneficial effects have been reported following RSV treatment of patients that were either healthy, obese or presenting different pathologies associated with the metabolic syndrome, other studies reported a lack of effects of this molecule (discussed by [6]).

One fact could potentially explain the apparent paradox of a molecule with low biological availability providing pleiotropic beneficial effects in many different contexts: it is that RSV can globally change the composition of endogenous microRNA populations [13]. MicroRNAs are small (19–23-nt in length) non-coding regulatory RNAs that regulate the stability and/or translation of their target transcripts. MicroRNAs have been progressively implicated in all aspects of cell biology and homeostasis and established as key players in a number of pathologies, including, but not limited to, inflammatory, metabolic and cardiovascular diseases, neuropathologies, and cancers [14,15,16,17,18,19,20,21,22,23,24,25,26].

## 2. Resveratrol-Inducible *MiR-663* in Health and Disease

MicroRNAs that regulate fundamental functions, such as metabolism, cell proliferation and differentiation, or development, have generally been very well conserved between invertebrates and vertebrates during evolution. In contrast, other microRNAs are found in vertebrates only: thus, *miR-155*, a microRNA that has been implicated in inflammatory response and inflammation-associated cancers. Finally, some microRNAs are only found in one or a few species of vertebrates. This is the case for *miR-663* (a.k.a. *miR-663a*) that appears to be primate-specific. A few years ago, it was shown that, upon RSV treatment, *miR-663* was upregulated both in human THP-1 monocytes, where it targets transcripts encoding pro-inflammatory JunB and JunD, and in human SW480 colon cancer cells, where it targets *TGFβ1* transcripts [27,28] (Table 1). At first, this suggested that this microRNA by and large would deliver beneficial effects to the body. Nevertheless, available literature shows that this is not always the case (see here after), indicating that *miR-663* can be harmful as well, depending on the cellular context.

### 2.1. MiR-663 in Inflammation

The capability of RSV to modulate a wide range of signaling pathways implicated in both the mounting and the termination of the immune response have puzzled scientists for a long time. It is becoming increasingly clear that many of these effects of RSV are through the modification of the composition of microRNA populations within the cell [13,29]. Namely, microRNAs are global regulators with the capability to directly regulate tens to hundreds of target transcripts, and many more through indirect regulation that results from microRNAs targeting transcripts encoding regulators such as transcription factors, factors implicated in different signal transduction pathways, such as kinases or phosphatases, or epigenetic regulators such as methylases or demethylases.

For example, *miR-663* has been shown to decrease AP-1 activity, that is critical for the mounting of the inflammatory response, by directly targeting *JunB* and *JunD* transcripts, at least in part through the downreguation of *miR-155* [27]. In addition, RSV impairs the up-regulation of pro-inflammatory *miR-155* at least in part through increasing *miR-663* expression [27]. This property of RSV is likely to have major consequences, for *miR-155* is implicated in the mounting of both the innate and adaptative immune responses [16,30]. A study conducted on 35 hypertensive patients with coronary artery disease and type 2 diabetes showed that, after ingesting a grape extract containing RSV for one year, patients presented with peripheral blood mononuclear cells that expressed less pro-inflammatory IL-1β and TNFα cytokines, less pro-inflammatory *miR-155*, and more anti-inflammatory *miR-663*. Although conducted on a relatively small sample of patients, this study provides a good evidence that long lasting, low RSV doses have actual beneficial effects on patient health [31]. Incidentally, it further suggests that RSV might be a better candidate for preventive rather than for curative therapeutics, at least when it comes to inflammation-related pathologies.

In contrast, in patients with systemic lupus erythematous, *miR-663* impairs the proliferation and migration of bone marrow-derived mesenchymal stem cells, thus shifting the imbalance between follicular T helper cells and regulatory T cells toward less regulatory T cells and more follicular T helper cells. As regulatory T cells reduce the capability of B cells to augment autoimmunity, *miR-663* activity, by reducing the secretion of *TGFβ1* by T cells, worsens lupus development [32]. In addition, *miR-663* activity proved deleterious in rheumatoid arthritis, a disease linked to synovial inflammation, cartilage erosion and joint destruction. In fibroblast-like synoviocytes from rheumatoid arthritis patients, increased levels of *miR-663* suppressed the expression of *Adenomatous polyposis coli* (*APC*) gene, triggering the activation of the canonical Wnt signaling pathway through accumulation of β-catenin. This activation increases the production of pro-inflammatory cytokines and, as a consequence of increased inflammation in joints, disturb the osteoblast–osteoclast axis and increases bone resorption by osteoclasts [33].

Nuclear factor TDP-43 (trans-activation response element DNA-binding protein 43) is an RNA-binding protein that shuttles between the nucleus and the cytoplasm. TDP-43 in particular plays a role in the biogenesis of microRNAs through its interactions with the nuclear Drosha complex, which generates pre-miRNAs from pri-miRNAs, and the cytoplasmic Dicer complex, which then produces mature miRNAs from pre-miRNAs [34]. TDP-43 has been shown to be causative in amyotrophic lateral sclerosis (ALS) and frontotemporal dementia (FTD) [35]. Interestingly, TDP-43 can bind microRNAs such as *let-7b* and *miR-663*. *TDP-43* knockdown in cultured cells decreases *let-7b* levels while increasing that of *miR-663*, thus modulating their activities [36]. It is thus possible that the role played by TDP-43 in neurodegenerative pathologies might be linked to its differential effects on microRNA production, and ultimately depends on whether these microRNAs are beneficial or deleterious in the context.

Finally, radiation-induced bystander effect corresponds to the biological response to radiations of cells that, while not being located in the path of ionizing rays, receive signals produced by directly irradiated cells that lead them to amplify or exaggerate the action of low dose radiation. This effect can significantly increase radiation risk and tissue damage. In particular, *TGFβ1* secretion by directly irradiated Hela cells reduces the expression of *miR-663* in both directly irradiated and bystander Hela cells, which correlates with increased DNA damage and reduced rate of cell survival. At the same time, *TGFβ1* signaling increases the expression of *miR-663* in bystander cells, which in turn decreases the levels of *TGFβ1* by targeting *TGFβ1* transcripts. By reducing *TGFβ1*-induced DNA damage, *miR-663* increases the survival of bystander cells, thus limiting the propagation of radiation-induced bystander effects [37]. Hence, the study of the effects of a low irradiation dose provides a further evidence for *miR-663* activity being at the same time beneficial as well as deleterious.

Therefore, the functions of *miR-663* seem to be context-dependent: it may well be that, in a given setting, the outcome of *miR-663* activity might depend on the context, i.e., on the transcriptome expressed in a given cell, and especially the panel of *miR-663* target transcripts that are present. It is also most probable that *miR-663* effects might be dose-dependent, and that it might target different transcripts as it has previously been shown for several microRNAs. For example, *miR-155* targeting of *Quaking* transcripts in RAW264.7 macrophages occurred at low concentration only [38], and, in myeloid cell from patients with acute myeloid leukemia, *miR-155* activity increases and decreases the levels of different set of transcripts depending of the level of *miR-155* expression [39]. Of note, RSV has the capability to change the level of expression of both *miR-155* and *miR-663* microRNAs, in both cases with apparent beneficial outcome [26,27]. Many other pathologies have also been associated with high levels of *miR-155*. For example, this microRNA has recently been shown to be causative in paralysis that develops following thoracic abdominal aortic aneurysm repair [40]. On the other hand, increased expression of chromosome 21-located *miR-155* in the brain of individuals with Down’s dementia has been linked with the presence of hyperphosphorylated tau protein and the reduction of the levels of several *miR-155* targets, including BACH1, CoREST1, BCL6, BIM, BCL10, Cyclin D, and SAPK4 [41]. Therefore, the capability of *miR-663* to decrease *miR-155* expression [29] and reported capability of RSV to cerebral ischemia, in particular through its anti-inflammatory effects, [9] suggests that this compound might be protective when administered ahead of programmed surgery or intervention.

### 2.2. MiR-663 in Cancer

It is now recognized that microRNAs play a central role in molecular dysfunctions linking inflammation with cancer [42,43]. While certain microRNAs are generally considered as pro-oncogenic or oncomiRs and others as tumor-suppressors, it seems that their actual impact on cancers might be context- and/or dose-dependent. This is in particular the case for *miR-155*, that is implicated in the mounting of a robust anti-tumor immunity when expressed at high doses but favors tumorigenesis when expressed at moderate level [39,42,43]. More generally, changes in microRNA expression either are causative in the initiation of cancers, or a consequence of the process of tumorigenesis itself. Remarkably, *miR-155* displays mutator activity, in particular due to its targeting of transcripts encoding the cell-cycle regulator WEE1 [44].

As seen here above for inflammation, *miR-663* can either inhibit or favor cell proliferation and/or migration in different settings. In human MCF7 breast cancer cells, *miR-663* targets transcripts encoding Eukaryotic translation elongation factor 1A2 (eEF1A2), which results in slowing the proliferation of MCF7 cells. RSV treatment of these cells increased the expression of *miR-663* and *miR-744*, with a similar output [45]. Breast cancer has a higher incidence in young Lebanese women as compared with American woman. A comparative profiling study showed that, in Lebanese breast cancer patients, 21 miRNAs, including *miR-663*, were specifically deregulated, possibly as a result of differential methylation of their promoter [46]. Another study showed that *miR-663* is up-regulated in multidrug-resistant MDA-MB-231-derived ADM cell line, and that increased *miR-663* expression was associated with the downregulation of Heparin sulfate proteoglycan 2 (HSPG2) and chemoresistance [47].

*MiR-663* was also shown to increase the proliferation of nasopharyngeal carcinoma NPC C666-1 cells by directly targeting the cell cycle negative regulator CDKN2A [48]. Accordingly, *miR-663* expression was higher in the serum of nasopharyngeal carcinoma patients, as compared with controls, and increasing *miR-663* levels were correlated with malignant progression and poor prognosis. On the other hand, *miR-663* expression was decreased by chemoradiotherapy [49]. The oncogenic activity of *miR-663* in nasopharyngeal carcinoma was due to its targeting of p21(WAF1/CIP1) that promotes the cellular G1/S transition [50]. In contrast, in two papillary thyroid carcinoma cell lines, *miR-663* behaved as a tumor-suppressor by targeting *TGFβ1*, thus inhibiting epithelium-to-mesenchyme transition [51], similar to what was previously found in SW480 colon cancer cells [28]. Similarly, *miR-663* levels were low in several human gastric cancer cell lines, and transfecting the two human gastric cancer cell lines BGC823 and SNU5 with *miR-663* suppressed their proliferation and induced a phenotype of mitotic catastrophe, indicating that *miR-663* behaves as a tumor-suppressor in this type of cancer [52]. A study about the effects of sunitinib treatment of metastatic renal cell carcinoma patients showed that the resistance that these patients develop eventually is linked to the downregulation of *miR-1* and *miR-663*. This downregulation was associated with the acquisition of a migratory phenotype, as established on xenografts. In sunitinib resistant tumor cells, *miR-663* targets *FRAS1* (Fraser Extracellular Matrix Complex Subunit 1) and *MDGA1* (MAM Domain Containing Glycosylphosphatidylinositol Anchor 1) transcripts. Restoring *miR-1* and *miR-663* levels or knocking down MDGA1 decreased renal cancer cell proliferation and migration [53]. The expression of *miR-663* was upregulated in HepG2 hepatocellular carcinoma cells co-incubated with the endoplasmic reticulum stress inducer tunicamycin. In these cells, *miR-663* inhibited apoptosis induced by endoplasmic reticulum stress by targeting *TGFβ1* transcripts [54]. In agreement with this result, *miR-663* was one of seven microRNAs whose expression was specifically changed in patients with hepatitis B virus-related HCC [55]. In pancreatic cancer tissues and cell lines, the downregulation of *miR-663* inversely correlated with the upregulation of transcipts encoding eEF1A2. eEF1A2 and *miR-663* levels were linked with TNM (tumor/node/metastasis) stage and node metastasis status in the patients. *MiR-663* was shown to decrease the proliferation and invasion potentials of pancreatic cancer cells both in vitro and in vivo by directly targeting *eEF1A2* [56]. A study about colorectal cancer, based on 109 biopsy specimens, compared biopsied from patients with tubulovillous adenomas and high-grade dysplasia versus biopsies from patients with normal mucosa or hyperplastic polyps. It showed that, among 99 microRNAs whose expression was different between the two groups, *miR-663*, *miR-1268*, *miR-320b*, *miR-1275*, and *miR-320b* were the most upregulated microRNAs in the biopsies of the first group of patients [49]. *MiR-663* was also upregulated in biopsies from cutaneous tissues with malignant melanoma [57].

Patients with lung cancers present with high level of *miR-663* expression, and *miR-663* direct or indirect targeting of *TGFβ1*, *P53*, *Bax*, and *Fas* transcripts increased the proliferation of A549 lung cancer cells [58]. Accordingly, *miR-663* proved deleterious in non-small cell lung cancer cells by targeting *PUMA/BBC3* (p53 up-regulated modulator of apoptosis/Bcl-2 binding component 3) and *BTG2* (B-cell translocation gene 2) transcripts, thus allowing cancer cells to escape apoptosis and promoting tumor onset and development [59]. Nevertheless, waltonitone treatment inhibited proliferation and induced apoptosis of H460 and H3255 lung cancer cell lines at least in part through inducing the targeting of *Bcl-2* by *miR-663* [60]. These results provide a further illustration of the ambiguous role *miR-663* can play in a particular type of tumor.

In a study on glioblastomas, the most aggressive brain tumor, the grade of tumors correlated with the level of PI3KCD activity (phosphatidylinositol-4,5-bisphosphate 3-kinase catalytic subunit delta) but inversely correlated with the level of *miR-663* expression. Higher *miR-663* levels were associated with increased patient survival, which was linked with *miR-663* targeting transcripts encoding PI3KCD [61]. *MiR-663* was also shown to decrease glioblastoma by targeting transcripts encoding CXCR4, the receptor of chemokine CXCL12 that is known to be involved in glioblastoma progression, and *miR-663* overexpression prolonged survival of mice with glioblastoma [62]. *MiR-663* was downregulated in A172 and U87 glioblastoma cell lines. Transfecting these cells with *miR-663* inhibited their proliferation, migration and invasion. These effects were through *miR-663* direct targeting of *TGFβ1*, as well as transcripts encoding *TGFβ1* downstream mediators MMP2 (Matrix metalloprotease 2) and E-cadherin [63]. On the other hand, *miR-663* expression was upregulated in castration-resistant prostate cancer tissues, and *miR-663* overexpression in LNCaP prostate cancer cells increased their potentials for proliferation, invasion and neuroendocrine differentiation, while reducing dihydrotestosterone-induced upregulation of prostate-specific antigen expression. In situ hybridization experiments established that the level of expression of *miR-663* correlates with TNM stage and Gleason score and is a good predictor of cancer recurrence [64]. In a meta-analysis, STAT3 (Signal Transducers and Activators of Transcription 3), JUN and JUNB transcription factors were identified as key signatures of a metastatic integrative regulatory network in prostate cancer progression. *MiR-663*, that is overexpressed in these types of cancers, was one of five microRNAs responsible for the down-regulation of the genes encoding these three transcription factors [65]. *MiR-663*, along with *miR-622* and *miR-647*, was upregulated in Taxol-resistant ovarian cancer cells, and the survival of Taxol-resistant patients with lower levels of *miR-663* and *miR-622* expression was significantly longer than patients with higher levels of expression of these two microRNAs [66].

As for liquid malignancies, *miR-663* was downregulated in K-562 cell line and in the white blood cells of certain patients with chronic myelogenous leukemia, due to the aberrant methylation of CpG islands upstream of *miR-663* gene. *MiR-663* suppressed K-562 cell proliferation at least in part through the targeting of *H-ras* transcripts [67]. The promoter of *miR-663* was also found to be hypermethylated in Chinese pediatric acute myeloid leukemia [68]. *MiR-663* was one of nine microRNAs most constantly upregulated in multiple myeloma cell lines. The upregulation of these microRNAs, including *miR-663* and *miR-155*, was linked with decreased viability, migration and colony formation of these cell lines. In addition, the higher expression levels of these microRNAs correlated with better patient survival [69]. Thus, *miR-663* behaves as a tumor-suppressor in liquid malignancies, in agreement with previous results showing that *all-trans* retinoic acid, a powerful pro-differentiation agent, induces the differentiation of HL-60 acute myeloid leukemia cells through the up-regulation of *miR-663* [70].

Epigenetic deregulation of *miR-663* expression appears to be a rather general feature of liquid malignancies. Of note, it was recently shown that the regulation of *miR-663* expression through epigenetic modification of its promoter depends of mitochondria-to-nucleus retrograde signaling, as shown by the downregulation of *miR-663* in cells lacking mitochondria, and also that *miR-663* mediates mitochondria-to-nucleus retrograde signaling [71]. Mitochondrial impairment through pharmacological disruption of oxidative phosphorylations, that increases reactive oxygen species, reduces *miR-663* expression. *MiR-663* regulates the expression of nuclear-encoded respiratory chain subunits involved in Complexes I, II, III, and IV, as well as that of Complexes I (NDUFAF1), II (SDHAF2), III (UQCC2), and IV (SCO1) assembly factor. In particular, *miR-663* activity is required for stabilizing respiratory supercomplexes, and directly regulated *UQCC2* expression. Mitochondrial dysfunction is one of the hallmarks of cancer, and indeed *miR-663* ectopic expression decreased tumor weight in xenografts and decreased cellular invasiveness of MCF7 and MDA-MB-231 breast cancer cell lines [71].

In conclusion, *miR-663* can either promote or inhibit tumorigenesis and metastasis depending on the context and the type of tumors. It remains to be shown whether it could be possible to turn *miR-663* from deleterious to beneficial in tumors where its activity correlates with increased tumorigenesis, rather than trying to inhibit its expression. RSV could be a good candidate compound, given its established anti-proliferation and anti-tumors effects associated with it capability to modulate the expression of *miR-663* along with that of other microRNAs. Of note, RSV effects in different cancers may have epigenetic bases, given its capacity to modulate the expression of NAD^+^-dependent histone deacetylases (Sirtuins), and particularly to activate Sirt1 and Sirt5 while inhibiting Sirt3 activity [72].

### 2.3. MiR-663 in Atherosclerosis

Atherosclerosis is a chronic inflammatory disease of the vascular wall, which, if left unchecked, turns into pathologies such as myocardial infarction and ischemic stroke, that are some the most prevalent causes of morbidity and lethality in the most developed countries. Atherosclerosis, although associated with systemic risk factors such as hypercholesterolemia, hypertension, and diabetes mellitus, most usually initiates in regions exposed to disturbed blood flow (d-flow), while arterial regions exposed to stable flow (s-flow) remain healthy [73]. It has been established that d-flow induces, and s-flow prevents endothelial dysfunction and atherosclerosis, respectively, at least in part through alterations in gene expression associated with changes in the epigenetic landscape [73]. Among these genes, microRNAs have been classified into three categories depending on their effects on atherogenesis: antiatherogenic mechano-miRs, proatherogenic mechano-miRs, and dual-role mechano-miRs [73].

*MiR-663* was identified among microRNAs that were upregulated in umbilical vein endothelial cells (HUVECs) submitted to oscillatory shear stress. In these cells, *miR-663* was implicated in monocyte adhesion but not in apoptosis [74]. It was subsequently shown that, in endothelial cells, *miR-663* plays a role in the upregulation of the gene encoding the transcription factor ATF4 and of its downstream gene VEGF, as well as in the activation of the ATF4 branch of unfolded protein response by oxidized phospholipids [75]. It was further shown that high concentrations of uric acid inhibit endothelial cell migration by upregulating *miR-663*, that directly targets *TGFβ1*. Higher *miR-663* levels were also found in the serum of hyperuricemic patients and animals [76]. Nevertheless, *miR-663* was shown to inhibit vascular smooth muscular cell phenotypic switch (the transformation from a contractile, differentiate phenotype to a synthetic, dedifferentiated phenotype associated with artery injury) by targeting *JUNB* and *MYH9* (myosin light chain 9 expression) transcripts [77]. *MiR-663* was further implicated in the induction of atherosclerosis by *Helicobacter pylori* [78].

Finally, as previously mentioned, a study conducted on peripheral blood mononuclear cells of type 2 diabetes and hypertensive patients with coronary showed that one-year supplementation with a grape extract containing RSV modulates the expression of inflammation-related microRNAs and cytokines expression [31]. In particular, pro-inflammatory *miR-155* was down-regulated, while *miR-663* was up-regulated, with correlative downregulation of *JUND* [31], a validated target of *miR-663* [27].

## 3. Conclusions

The literature analyzed here above clearly demonstrates that it goes for microRNAs as it goes for coding genes: none of them is just only detrimental, none of them is capable of producing beneficial effects in all circumstances. Thus, the analysis of molecular effects and functions of this class of regulators requires a rather expressionist approach, looking for bright effects aside the deleterious ones. This is in particular the case for *miR-663*, that, as seen in Table 1, depending on the context and the type of cells and tumors considered, can be either pro- or anti-inflammatory, or behave either as a tumor-suppressor gene or well favor tumorigenesis. Also, while *miR-663* has been several times described as a “bad” microRNA, it nevertheless seems capable to deliver tumor-suppressive effects and to prevent vascular smooth muscular cell phenotypic switch that is associated with arterial injury.

Although microRNAs can successively, or possibly at the same time, behave as the bad, the good or the ugly, like the heroes of the classical Western movie with a similar name, one impressive conclusion that can be drawn from the study of the effects of RSV on *miR-663* expression is that this biological compound has the remarkable propriety to induce the good behavior while inhibiting the bad and the ugly ones. Thus, it has been previously shown that RSV treatment of SW480 colon cancer cells leads to both the downregulation of microRNAs known to favor cancer initiation and progression and the upregulation of microRNAs usually considered as tumor-suppressors, including *miR-663* in this context [28]. In this respect, it should be noted that both, *miR-663* and RSV are implicated in the regulation of the *TGFβ1* signaling pathway. This might possibly explain at least in part why RSV can deliver beneficial effects trough the modulation of *miR-663* expression, given that *TGFβ1* can be cytostatic at the early stages of cancer while also favoring epithelium-to-mesenchyme transition at more advances stages of tumorization, owing to the similar function it plays during development. In addition, *TGFβ1* is also implicated in the regulation of the immune response, and systemic immune suppression and inhibition of host immunosurveillance favors cancer development [79]. Furthermore, it has been shown that *TGFβ1* up-regulates *miR-155* in hepatocellular carcinoma cells, thus promoting epithelium-to-mesenchyme transition, invasion and metastasis [80], and that *miR-155* plays a role in mediating *TGFβ1*-induced podocyte injury via nephrin, desmin and caspase-9 [81]. Therefore, the capability of RSV to modulate, directly or indirectly, the levels of *miR-155*, *miR-663* and *TGFβ1* activity, and the fact that the three last molecules display dose-dependent activity and can be either beneficial or deleterious to the body, depending on the context, may possibly explain the apparent pleiotropic beneficial effects of RSV, and certainly warrants further studies.

Given that RSV seems to be active while provided at low dose for a sustained period, rather than at higher doses for a shorter period, it is possible that the wide range of beneficial properties of this plant polyphenol may rely on its capacity to simultaneously reset the expression of multiple microRNAs within a range of concentrations where they would work for the health of the organism, rather than just sharply increasing or decreasing their expression. This might possibly explain RSV apparent lack of delivering increasingly beneficial effects at increasing doses, a fact that led to suggestion that most of RSV apparent properties may rather result from experimental artifacts. Beside all its proved or potential beneficial effects to the health of the individual, RSV may well provide us with a new tool for the study of dose-dependent activity of microRNAs and other non-coding regulatory RNAs in the future.

## Figures and Tables

**Table 1:** Validated and putative target transcripts of *miR-663* that link this microRNA with inflammatory, neurodegenerative and cardiovascular diseases, as well as with cancer.

Target Transcripts	*MiR-663* Effects	References
**Opposite effects on inflammation**		
*JunB*, *JunD*	Anti-inflammatory, through reducing AP-1 activity and *miR-155* expression	[27]
*TGFβ1*	Worsens lupus erythematous development	[32]
*APC*	Pro-inflammatory through the activation of Wnt pathway	[33]
**Stimulation of cell proliferation and migration**		
*TGFβ1*	Increases the survival of non-irradiated bystander cells	[37]
*HSPG2*	Increases chemoresistance of MDA-MB-231/ADM cell line	[47]
*TGFβ1*	Inhibits apoptosis induced by endoplasmic reticulum stress	[54]
*TGFβ1*, *P53*, *Bax*, *Fas*	Increases lung cancer cell proliferation	[57]
*PUMA/BBC3*, *BTG2*	Inhibits apoptosis and promotes tumor development	[59]
**Inhibition of cell proliferation and migration**		
*eEF1A2*	Impairs the proliferation of MCF7 cells	[45]
*CDKN2A*	Promotes the proliferation of nasopharyngeal carcinoma C666-1 cells	[48]
*p21*(*WAF1/CIP1*)	Promotes the proliferation of nasopharyngeal carcinoma cells	[50]
*TGFβ1*	Antimetastatic in SW480 colorectal cancer cells	[28]
*TGFβ1*	Inhibits epithelium-to-mesenchyme transition of two thyroid carcinoma cell lines	[51]
*MDGA1*, *FRAS1*	decreases renal cancer cell proliferation and migration	[53]
*eEF1A2*	Inhibits proliferation and invasion of pancreatic cancer cells	[56]
*Bcl-2*	Implicated in waltonitone treatment-induced inhibition of lung cancer cell line proliferation	[60]
*PI3KCD*	Inhibits proliferation and invasiveness of glioblastoma cells	[61]
*CXCR4*	Increases survival of mice with glioblastoma	[62]
*TGFβ1*, *MMP2*, *E-Cadherin*	Inhibits proliferation and invasiveness of glioblastoma cells	[63]
*H-ras*	Inhibits proliferation of K-562 cells	[67]
*UQCC2*	Increases phosphorylative oxidations and decreases tumor development	[72]
**Prevention of arterial injury**		
*TGFβ1*	Inhibits endothelial cell migration under high concentrations of uric acid	[76]
*JUNB*, *MYH9*	Inhibits vascular smooth muscular cell phenotypic switch	[77]

## References

[1] Delmas D., Lançon A., Colin D., Jannin B., Latruffe N. (2006). Resveratrol as a chemopreventive agent: A promising molecule for fighting cancer. Curr. Drug Targets.

[2] Tili E., Michaille J.-J. (2011). Resveratrol, MicroRNAs, Inflammation, and Cancer. J. Nucleic Acids.

[3] Sinha D., Sarkar N., Biswas J., Bishayee A. (2016). Resveratrol for breast cancer prevention and therapy: Preclinical evidence and molecular mechanisms. Semin. Cancer Biol..

[4] Bartolacci C., Andreani C., Amici A., Marchini C. (2018). Walking a Tightrope: A Perspective of Resveratrol Effects on Breast Cancer. Curr. Protein Pept. Sci..

[5] Zordoky B.N., Robertson I.M., Dyck J.R. (2015). Preclinical and clinical evidence for the role of resveratrol in the treatment of cardiovascular diseases. Biochim. Biophys. Acta.

[6] Erdogan C.S., Vang O. (2016). Challenges in analyzing the biological effects of resveratrol. Nutrients.

[7] Singh C.K., Ndiaye M.A., Ahmad N. (2015). Resveratrol and cancer: Challenges for clinical translation. Biochim. Biophys. Acta.

[8] Ko J.H., Sethi G., Um J.Y., Shanmugam M.K., Arfuso F., Kumar A.P., Bishayee A., Ahn K.S. (2017). The Role of Resveratrol in Cancer Therapy. Int. J. Mol. Sci..

[9] Lee R.H.C., Lee M.H.H., Wu C.Y.C., Couto e Silva A., Possoit H.E., Hsieh T.H., Minagar A., Lin H.W. (2018). Cerebral ischemia and neuroregeneration. Neural Regen. Res..

[10] Rauf A., Imran M., Suleria H.A.R., Ahmad B., Peters D.G., Mubarak M.S. (2017). A comprehensive review of the health perspectives of resveratrol. Food Funct..

[11] Bonnefont-Rousselot D. (2016). Resveratrol and Cardiovascular Diseases. Nutrients.

[12] Fogacci F., Tocci G., Presta V., Fratter A., Borghi C., Cicero A.F.G. (2018). Effect of resveratrol on blood pressure: A systematic review and meta-analysis of randomized, controlled, clinical trials. Crit. Rev. Food Sci. Nutr..

[13] Tili E., Michaille J.-J. (2016). Promiscuous Effects of Some Phenolic Natural Products on Inflammation at Least in Part Arise from Their Ability to Modulate the Expression of Global Regulators, Namely microRNAs. Molecules.

[14] Neudecker V., Yuan X., Bowser J.L., Eltzschig H.K. (2017). MicroRNAs in mucosal inflammation. J. Mol. Med..

[15] Tili E., Michaille J.-J., Piurowski V., Rigot B., Croce C.M. (2017). MicroRNAs in intestinal barrier function, inflammatory bowel disease and related cancers-their effects and therapeutic potentials. Curr. Opin. Pharmacol..

[16] Alivernini S., Gremese E., McSharry C., Tolusso B., Ferraccioli G., McInnes I.B., Kurowska-Stolarska M. (2018). MicroRNA-155-at the Critical Interface of Innate and Adaptive Immunity in Arthritis. Front. Immunol..

[17] Momen-Heravi F., Bala S. (2018). miRNA regulation of innate immunity. J. Leukoc. Biol..

[18] Curtale G. (2018). MiRNAs at the Crossroads between Innate Immunity and Cancer: Focus on Macrophages. Cells.

[19] Mirra P., Nigro C., Prevenzano I., Leone A., Raciti G.A., Formisano P., Beguinot F., Miele C. (2018). The Destiny of Glucose from a MicroRNA Perspective. Front. Endocrinol..

[20] Wojciechowska A., Braniewska A., Kozar-Kamińska K. (2017). MicroRNA in cardiovascular biology and disease. Adv. Clin. Exp. Med..

[21] Vogiatzi G., Oikonomou E., Deftereos S., Siasos G., Tousoulis D. (2018). Peripheral artery disease: A micro-RNA-related condition?. Curr. Opin. Pharmacol..

[22] Miya Shaik M., Tamargo I.A., Abubakar M.B., Kamal M.A., Greig N.H., Gan S.H. (2018). The Role of microRNAs in Alzheimer’s Disease and Their Therapeutic Potentials. Genes.

[23] Narayan N., Bracken C.P., Ekert P.G. (2018). MicroRNA-155 expression and function in AML: An evolving paradigm. Exp. Hematol..

[24] Farooqi A.A., Khalid S., Ahmad A. (2018). Regulation of Cell Signaling Pathways and miRNAs by Resveratrol in Different Cancers. Int. J. Mol. Sci..

[25] Vannini I., Fanini F., Fabbri M. (2018). Emerging roles of microRNAs in cancer. Curr. Opin. Genet. Dev..

[26] Ramassone A., Pagotto S., Veronese A., Visone R. (2018). Epigenetics and MicroRNAs in Cancer. Int. J. Mol. Sci..

[27] Tili E., Michaille J.-J., Adair B., Alder H., Limagne E., Taccioli C., Ferracin M., Delmas D., Latruffe N., Croce C.M. (2010). Resveratrol decreases the levels of miR-155 by upregulating miR-663, a microRNA targeting JunB and JunD. Carcinogenesis.

[28] Tili E., Michaille J.-J., Alder H., Volinia S., Delmas D., Latruffe N., Croce C.M. (2010). Resveratrol modulates the levels of microRNAs targeting genes encoding tumor-suppressors and effectors of *TGFβ* signaling pathway in SW480 cells. Biochem. Pharmacol..

[29] Latruffe N., Lançon A., Frazzi R., Aires V., Delmas D., Michaille J.-J., Djouadi F., Bastin J., Cherkaoui-Malki M. (2015). Exploring new ways of regulation by resveratrol involving miRNAs, with emphasis on inflammation. Ann. N. Y. Acad. Sci..

[30] Tili E., Michaille J.-J., Cimino A., Costinean S., Dumitru C.D., Adair B., Fabbri M., Alder H., Liu C.G., Calin G.A. (2007). Modulation of miR-155 and miR-125b levels following lipopolysaccharide/TNF-alpha stimulation and their possible roles in regulating the response to endotoxin shock. J. Immunol..

[31] Tomé-Carneiro J., Larrosa M., Yáñez-Gascón M.J., Dávalos A., Gil-Zamorano J., Gonzálvez M., García-Almagro F.J., Ruiz Ros J.A., Tomás-Barberán F.A., Espín J.C. (2013). One-year supplementation with a grape extract containing resveratrol modulates inflammatory-related microRNAs and cytokines expression in peripheral blood mononuclear cells of type 2 diabetes and hypertensive patients with coronary artery disease. Pharmacol. Res..

[32] Geng L., Tang X., Zhou K., Wang D., Wang S., Yao G., Chen W., Gao X., Chen W., Shi S. (2018). MicroRNA-663 induces immune dysregulation by inhibiting *TGF-β1* production in bone marrow-derived mesenchymal stem cells in patients with systemic lupus erythematosus. Cell. Mol. Immunol..

[33] Miao C.G., Shi W.J., Xiong Y.Y., Yu H., Zhang X.L., Qin M.S., Du C.L., Song T.W., Zhang B., Li J. (2015). MicroRNA-663 activates the canonical Wnt signaling through the adenomatous polyposis coli suppression. Immunol. Lett..

[34] Kawahara Y., Mieda-Sato A. (2012). TDP-43 promotes microRNA biogenesis as a component of the Drosha and Dicer complexes. Proc. Natl. Acad. Sci. USA.

[35] Ling S.C., Polymenidou M., Cleveland D.W. (2013). Converging mechanisms in ALS and FTD: Disrupted RNA and protein homeostasis. Neuron.

[36] Buratti E., De Conti L., Stuani C., Romano M., Baralle M., Baralle F. (2010). Nuclear factor TDP-43 can affect selected microRNA levels. FEBS J..

[37] Hu W., Xu S., Yao B., Hong M., Wu X., Pei H., Chang L., Ding N., Gao X., Ye C. (2014). MiR-663 inhibits radiation-induced bystander effects by targeting *TGFβ1* in a feedback mode. RNA Biol..

[38] Tili E., Chiabai M., Palmieri D., Brown M., Cui R., Fernandes C., Richmond T., Kim T., Sheetz T., Sun H.L. (2015). Quaking and miR-155 interactions in inflammation and leukemogenesis. Oncotarget.

[39] Narayan N., Morenos L., Phipson B., Willis S.N., Brumatti G., Eggers S., Lalaoui N., Brown L.M., Kosasih H.J., Bartolo R.C. (2017). Functionally distinct roles for different miR-155 expression levels through contrasting effects on gene expression, in acute myeloid leukaemia. Leukemia.

[40] Awad H., Bratasz A., Nuovo G., Burry R., Meng X., Kelani H., Brown M., Ramadan M.E., Bouhliqah L., Popovich P.G. (2018). MiR-155 deletion reduces ischemia-induced paralysis in a TAAA repair mouse model. Ann. Diagn. Pathol..

[41] Tili E., Mezache L., Michaille J.-J., Amann V., Williams J., Vandiver P., Quinonez M., Fadda P., Mikhail A., Nuovo G. (2018). microRNA 155 up regulation in the CNS is strongly correlated to Down’s syndrome dementia. Ann. Diagn. Pathol..

[42] Tili E., Michaille J.-J., Croce C.M. (2009). miR-155: On the crosstalk between inflammation and cancer. Int. Rev. Immunol..

[43] Tili E., Michaille J.-J., Croce C.M. (2013). MicroRNAs play a central role in molecular dysfunctions linking inflammation with cancer. Immunol. Rev..

[44] Tili E., Michaille J.-J., Wernicke D., Alder H., Costinean S., Volinia S., Croce C.M. (2011). Mutator activity induced by microRNA-155 (miR-155) links inflammation and cancer. Proc. Natl. Acad. Sci. USA.

[45] Vislovukh A., Kratassiouk G., Porto E., Gralievska N., Beldiman C., Pinna G., El’skaya A., Harel-Bellan A., Negrutskii B., Groisman I. (2013). Proto-oncogenic isoform A2 of eukaryotic translation elongation factor eEF1 is a target of miR-663 and miR-744. Br. J. Cancer.

[46] Nassar F.J., Talhouk R., Zgheib N.K., Tfayli A., El Sabban M., El Saghir N.S., Boulos F., Jabbour M.N., Chalala C., Boustany R.M. (2017). microRNA Expression in Ethnic Specific Early Stage Breast Cancer: An Integration and Comparative Analysis. Sci. Rep..

[47] Hu H., Li S., Cui X., Lv X., Jiao Y., Yu F., Yao H., Song E., Chen Y., Wang M. (2013). The overexpression of hypomethylated miR-663 induces chemotherapy resistance in human breast cancer cells by targeting heparin sulfate proteoglycan 2 (HSPG2). J. Biol. Chem..

[48] Liang S., Zhang N., Deng Y., Chen L., Zhang Y., Zheng Z., Luo W., Lv Z., Li S., Xu T. (2017). miR-663 promotes NPC cell proliferation by directly targeting CDKN2A. Mol. Med. Rep..

[49] Tsikitis V.L., Potter A., Mori M., Buckmeier J.A., Preece C.R., Harrington C.A., Bartley A.N., Bhattacharyya A.K., Hamilton S.R., Lance M.P. (2016). MicroRNA Signatures of Colonic Polyps on Screening and Histology. Cancer Prev. Res..

[50] Yi C., Wang Q., Wang L., Huang Y., Li L., Liu L., Zhou X., Xie G., Kang T., Wang H. (2012). MiR-663, a microRNA targeting p21(WAF1/CIP1), promotes the proliferation and tumorigenesis of nasopharyngeal carcinoma. Oncogene.

[51] Wang Z., Zhang H., Zhang P., Dong W., He L. (2016). MicroRNA-663 suppresses cell invasion and migration by targeting transforming growth factor beta 1 in papillary thyroid carcinoma. Tumor Biol..

[52] Pan J., Hu H., Zhou Z., Sun L., Peng L., Yu L., Sun L., Liu J., Yang Z., Ran Y. (2010). Tumor-suppressive mir-663 gene induces mitotic catastrophe growth arrest in human gastric cancer cells. Oncol. Rep..

[53] Butz H., Ding Q., Nofech-Mozes R., Lichner Z., Ni H., Yousef G.M. (2017). Elucidating mechanisms of sunitinib resistance in renal cancer: An integrated pathological-molecular analysis. Oncotarget.

[54] Huang Y., Liu J., Fan L., Wang F., Yu H., Wei W., Sun G. (2016). miR-663 overexpression induced by endoplasmic reticulum stress modulates hepatocellular carcinoma cell apoptosis via transforming growth factor beta 1. OncoTargets Ther..

[55] Wang G., Dong F., Xu Z., Sharma S., Hu X., Chen D., Zhang L., Zhang J., Dong Q. (2017). MicroRNA profile in HBV-induced infection and hepatocellular carcinoma. BMC Cancer.

[56] Zang W., Wang Y., Wang T., Du Y., Chen X., Li M., Zhao G. (2015). miR-663 attenuates tumor growth and invasiveness by targeting eEF1A2 in pancreatic cancer. Mol. Cancer.

[57] Sand M., Skrygan M., Sand D., Georgas D., Gambichler T., Hahn S.A., Altmeyer P., Bechara F.G. (2013). Comparative microarray analysis of microRNA expression profiles in primary cutaneous malignant melanoma, cutaneous malignant melanoma metastases, and benign melanocytic nevi. Cell Tissue Res..

[58] Liu Z.Y., Zhang G.L., Wang M.M., Xiong Y.N., Cui H.Q. (2011). MicroRNA-663 targets TGFB1 and regulates lung cancer proliferation. Asian Pac. J. Cancer Prev..

[59] Fiori M.E., Villanova L., Barbini C., De Angelis M.L., De Maria R. (2018). miR-663 sustains NSCLC by inhibiting mitochondrial outer membrane permeabilization (MOMP) through PUMA/BBC3 and BTG2. Cell Death Dis..

[60] Zhang Y., Zhou X., Xu X., Zhang M., Wang X., Bai X., Li H., Kan L., Zhou Y., Niu H. (2015). Waltonitone induces apoptosis through mir-663-induced Bcl-2 downregulation in non-small cell lung cancer. Tumour Biol..

[61] Shi Y., Chen C., Zhang X., Liu Q., Xu J.L., Zhang H.R., Yao X.H., Jiang T., He Z.C., Ren Y. (2014). Primate-specific miR-663 functions as a tumor suppressor by targeting PIK3CD and predicts the prognosis of human glioblastoma. Clin. Cancer Res..

[62] Shi Y., Chen C., Yu S.Z., Liu Q., Rao J., Zhang H.R., Xiao H.L., Fu T.W., Long H., He Z.C. (2015). miR-663 Suppresses Oncogenic Function of CXCR4 in Glioblastoma. Clin. Cancer Res..

[63] Li Q., Cheng Q., Chen Z., Peng R., Chen R., Ma Z., Wan X., Liu J., Meng M., Peng Z. (2016). MicroRNA-663 inhibits the proliferation, migration and invasion of glioblastoma cells via targeting TGF-β1. Oncol. Rep..

[64] Jiao L., Deng Z., Xu C., Yu Y., Li Y., Yang C., Chen J., Liu Z., Huang G., Li L.C. (2014). miR-663 induces castration-resistant prostate cancer transformation and predicts clinical recurrence. J. Cell. Physiol..

[65] Sadeghi M., Ranjbar B., Ganjalikhany M.R., Khan F.M., Schmitz U., Wolkenhauer O., Gupta S.K. (2016). MicroRNA and Transcription Factor Gene Regulatory Network Analysis Reveals Key Regulatory Elements Associated with Prostate Cancer Progression. PLoS ONE.

[66] Kim Y.W., Kim E.Y., Jeon D., Liu J.L., Kim H.S., Choi J.W., Ahn W.S. (2014). Differential microRNA expression signatures and cell type-specific association with Taxol resistance in ovarian cancer cells. Drug Des. Devel. Ther..

[67] Yang Y., Wang L.L., Wang H.X., Guo Z.K., Gao X.F., Cen J., Li Y.H., Dou L.P., Yu L. (2013). The epigenetically-regulated miR-663 targets H-ras in K-562 cells. FEBS J..

[68] Yan-Fang T., Jian N., Jun L., Na W., Pei-Fang X., Wen-Li Z., Dong W., Li P., Jian W., Xing F. (2013). The promoter of miR-663 is hypermethylated in Chinese pediatric acute myeloid leukemia (AML). BMC Med. Genet..

[69] Bi C., Chung T.H., Huang G., Zhou J., Yan J., Ahmann G.J., Fonseca R., Chng W.J. (2015). Genome-wide pharmacologic unmasking identifies tumor suppressive microRNAs in multiple myeloma. Oncotarget.

[70] Jian P., Li Z.W., Fang T.Y., Jian W., Zhuan Z., Mei L.X., Yan W.S., Jian N. (2011). Retinoic acid induces HL-60 cell differentiation via the upregulation of miR-663. J. Hematol. Oncol..

[71] Carden T., Singh B., Mooga V., Bajpai P., Singh K.K. (2017). Epigenetic modification of miR-663 controls mitochondria-to-nucleus retrograde signaling and tumor progression. J. Biol. Chem..

[72] Schiedel M., Robaa D., Rumpf T., Sippl W., Jung M. (2018). The Current State of NAD^+^—Dependent Histone Deacetylases (Sirtuins) as Novel Therapeutic Targets. Med. Res. Rev..

[73] Kumar S., Kim C.W., Simmons R.D., Jo H. (2014). Role of flow-sensitive microRNAs in endothelial dysfunction and atherosclerosis: Mechanosensitive athero-miRs. Arterioscler. Thromb. Vasc. Biol..

[74] Ni C.W., Qiu H., Jo H. (2011). MicroRNA-663 upregulated by oscillatory shear stress plays a role in inflammatory response of endothelial cells. Am. J. Physiol. Heart Circ. Physiol..

[75] Afonyushkin T., Oskolkova O.V., Bochkov V.N. (2012). Permissive role of miR-663 in induction of VEGF and activation of the ATF4 branch of unfolded protein response in endothelial cells by oxidized phospholipids. Atherosclerosis.

[76] Hong Q., Yu S., Geng X., Duan L., Zheng W., Fan M., Chen X., Wu D. (2015). High Concentrations of Uric Acid Inhibit Endothelial Cell Migration via miR-663 Which Regulates Phosphatase and Tensin Homolog by Targeting Transforming Growth Factor-β1. Microcirculation.

[77] Li P., Zhu N., Yi B., Wang N., Chen M., You X., Zhao X., Solomides C.C., Qin Y., Sun J. (2013). MicroRNA-663 regulates human vascular smooth muscle cell phenotypic switch and vascular neointimal formation. Circ. Res..

[78] Kalani M., Hodjati H., GhamarTalepoor A., Samsami Dehaghani A., Doroudchi M. (2017). CagA-positive and CagA-negative Helicobacter pylori strains differentially affect the expression of micro RNAs 21, 92a, 155 and 663 in human umbilical vein endothelial cells. Cell. Mol. Biol..

[79] Yang L., Pang Y., Moses H.L. (2010). TGF-β and immune cells: An important regulatory axis in the tumor microenvironment and progression. Trends Immunol..

[80] Li D.P., Fan J., Wu Y.J., Xie Y.F., Zha J.M., Zhou X.M. (2017). MiR-155 up-regulated by TGF-β promotes epithelial-mesenchymal transition, invasion and metastasis of human hepatocellular carcinoma cells in vitro. Am. J. Trans. Res..

[81] Lin X., Zhen X., Huang H., Wu H., You Y., Guo P., Gu X., Yang F. (2017). Role of MiR-155 Signal Pathway in Regulating Podocyte Injury Induced by TGF-β1. Cell. Physiol. Biochem..

